# Sushrutha - our proud heritage

**Published:** 2009

**Authors:** Surajit Bhattacharya

**Affiliations:** Indian Journal of Plastic Surgery

Sushrutha is recognized today as the ‘Father of Plastic Surgery’ all over the world. The fact that such a man in flesh and blood performed these surgeries a millennium before Hippocrates and two millenniums before European stalwarts like Celsius and Galen ever appeared on the scene is hard for most to realize and appreciate.[1] Centuries of our mental subjugation and fascination for every occidental facet of life - culture, apparel, cuisine, architecture, science and medicine has made it difficult for us to explore and appreciate the original contributions of ancient pioneers of our own motherland. Sushrutha is one such shining star in our history, who continues to bedazzle one and all.

Sushrutha came from a rich heritage of learned scholars.[2] At the Sushrutha school, the first person to expound Āyurvedic knowledge was *Dhanvantari,* who taught it to *Divodasa,* who, in turn, taught it to Sushrutha, *Aupadhenava, Aurabhra, Paushakalāvata, Gopurarakshita,* and *Bhoja*. Sushrutha, a savant surgeon and philosopher, and above all a great teacher, compiled a monumental treatise in surgery, *Susrutasamhita.* He has considerable medical knowledge of relevance even today. It indicates that India was far ahead of the rest of the world in medical knowledge.

The *Susrutasamhita*[3] is in two parts, the *Purva-tantra* in five sections and the *Uttara-tantra*. The two parts together encompass, apart from *Salya* and *Salakya*, other specialities like medicine, paediatrics, geriatrics, diseases of the ear, nose, throat and eye, toxicology, aphrodisiacs and psychiatry. In the book's 184 chapters, 1,120 medical conditions, including injuries and illnesses relating to ageing and mental illness, are listed. The compendium of Sushrutha includes many chapters on the training and practice of surgeons. It also has 300 surgical procedures and classifies human surgery in eight categories. Sushrutha details 650 drugs of animal, plant, and mineral origin. It describes more than 300 kinds of operations that call for 42 different surgical processes and 121 different types of instruments.[3,4]

His *Samdamsa yantras* are the first forms of the modern surgeon's spring forceps and dissection and dressing forceps. In fact, his system of naming surgical tools after the animals or birds they resemble in shape, for example crocodile forceps, hawkbill forceps, is adopted even today. He was the first to introduce diagnostic instruments and their principles, which were modified later with the introduction of optical system in their construction to form the present day endoscopes. Fourteen types of bandaging capable of covering almost all the regions of the body were described for the practice of the student on dummies. The use of surgical devices such as tourniquets and setting plasters also find a mention. Some important procedures, which preceded actual surgery, such as cauterisation by *Ksaras* (alkaline substances) or *Agni* (fire – heat) and application of leeches were being practiced extensively by him.[3,5]

Sushrutha has covered accidental burns in its four degrees, the effect of heat-stroke, sun-stroke and frost-bite due to excessive cold and also the effect of lightning, which he calls *vidyut-dagdha*. This classification underlines his view that all thermogenic trauma, whether due to extreme cold or heat, either wet or dry, chemical or inert fluid, produces damage almost similar and hence has to be managed as one entity. The great value of Sushrutha's classification could be realized from the fact that this concept gained validity in modern surgery only recently after 1950 and is now uniformly accepted in the classification and management of these injuries.

Ancient surgical science was known as *Salya-tantra*.*Salya-tantra* (surgical science). It embraced all processes aiming at the removal of factors responsible for producing pain or misery to the body or mind. *Salya* (*salya*-surgical instrument) denotes broken parts of an arrow /other sharp weapons while *tantra* denotes manoeuvre. The broken parts of the arrows or similar pointed weapons were regarded as the commonest and most dangerous objects causing wounds and requiring surgical treatment. Shushrutha described surgery under eight heads *Chedya* (excision), *Lekhya* (scarification), *Vedhya* (puncturing), *Esya* (exploration), *Ahrya* (extraction), *Vsraya* (evacuation) and *Sivya* (Suturing).[6,7]

Sushrutha pointed out that haemorrhage could be arrested by apposition of the cut edges with stitches, application of styptic decoctions, by cauterisation with chemicals or heat. He advocated the use of wine with incense of cannabis for anaesthesia and there is no literature on anaesthesia anywhere in the world preceding this practice. Sushrutha thus can qualify to be called the ‘Father of Anaesthesiology’.

Sushrutha also gave classification of the bones and their reaction to injuries. Varieties of dislocation of joints (sandhimukta) and fractures of the shaft (*kanda-bhagna*) were described systematically in the *Samhita*. He classified and gave details of the six types of dislocations and 12 varieties of fractures. He offered the principles of fracture treatment, viz., traction, manipulation, appositions and stabilization. He described the entire trauma related orthopaedic surgery, including some measures of rehabilitation, in his celebrated work.[2]

He recognized diabetes and defined it as *Medhumeha*. He further explained it with obesity and sedentary lifestyle, advising exercises to help cure it. He also explains hypertension in a manner, which matches the modern symptoms of the disease. He related obesity, which was known to him, with diabetes and heart disorder. He recommended physical work to help cure it and its side effects. He was an expert in cataract surgery and had an elaborate knowledge of urinary stone disease.[8] His exploits in various aspects of urology and endoscopy have been illustrated by Das.[8,9]

Sushrutha was also an excellent teacher. He told his pupils that one could become a good physician only if one knew both theory and practice. He advised his pupils to use carcasses and models for practice before surgery. Sushrutha ordained that anyone who wants to attain surgical skill should study anatomy by practical observation of the various structures composing the body.

The study of anatomy is dealt with in the *Sarirasthana* of the *Sushrutasamhita*. He proposed to first deal with embryology and then anatomy of the human body, which he felt, was an extension of the embryo. He did not fail to warn that improper intervention with surgical manoeuvre due either to ignorance of the progress of the disease-process, greed for money or lack of judgment, lead only to complications. A conscientious surgeon, on the other hand, should consider his patient as a whole for diseases divorced from patients are abstractions from reality. Any surgical manoeuvre, he felt, was a phased programme planned well and then executed. His *pascatkarman* or post-operative schedule included the rehabilitation and removal of complications.[3]

Sushrutha took surgery in medieval India to admirable heights and that era was later regarded *The Golden Age of Surgery* in ancient India. Because of his numerous seminal contributions to the science and art of surgery in India, he is regarded as the ‘Father of Surgery’ and the ‘Father of Plastic Surgery’. In “The source Book of Plastic Surgery,” Frank McDowell aptly described Sushrutha as follows:

“Through all of Sushruta's flowery language, incantations and irrelevancies, there shines the unmistakable picture of a great surgeon. Undaunted by his failures, unimpressed by his successes, he sought the truth unceasingly and passed it on to those who followed. He attacked disease and deformity definitively, with reasoned and logical methods. When the path did not exist, he made one.”[10]

Surgery in India reached admirable heights during the era of Sushrutha. We are proud to have such a genius as the pioneer of our surgical heritage. The Association of Plastic Surgeons of India celebrates his life and time by giving him a proud position in the association's emblem!
